# Supplementary material for: Melanoma-Derived Exosomal miR-125b-5p Educates Tumor Associated Macrophages (TAMs) by Targeting Lysosomal Acid Lipase A (LIPA)
Source: Cancers (Basel). 2020 Feb 17;12(2):464. doi: 10.3390/cancers12020464 (PMC7072270; doi:10.3390/cancers12020464)
Supplement: Supplementary file 1 [file cancers-12-00464-s001.pdf]

Supplementary Figures

A

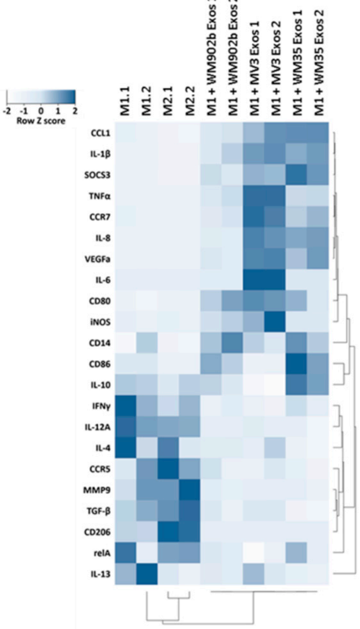

B

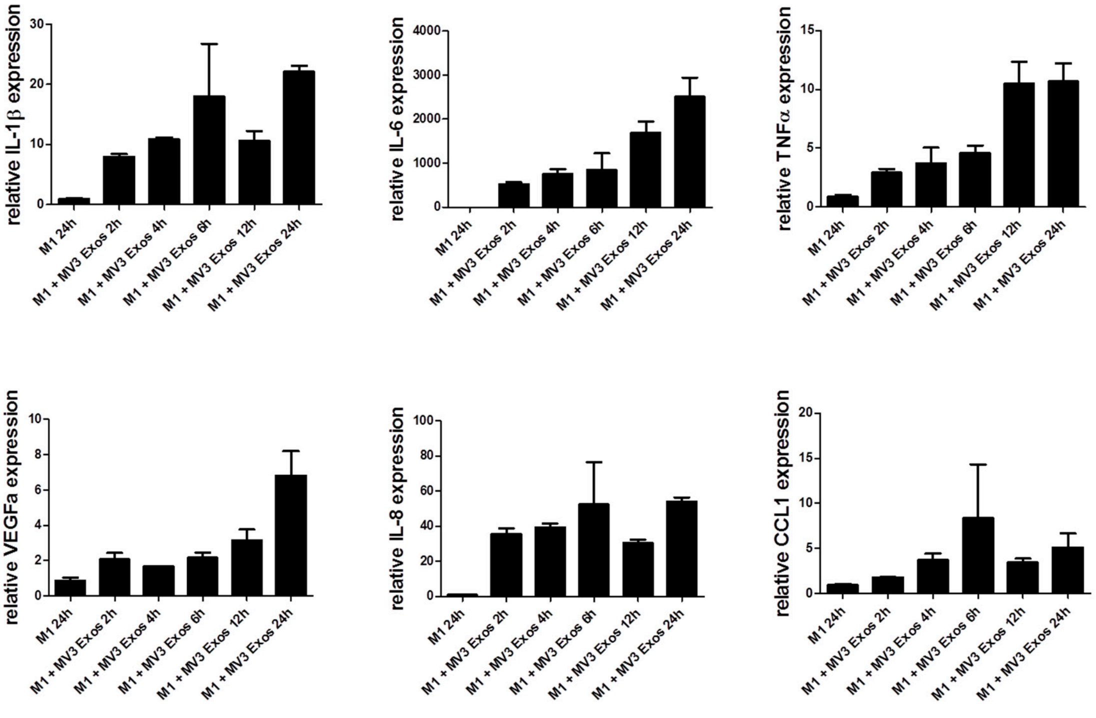

C

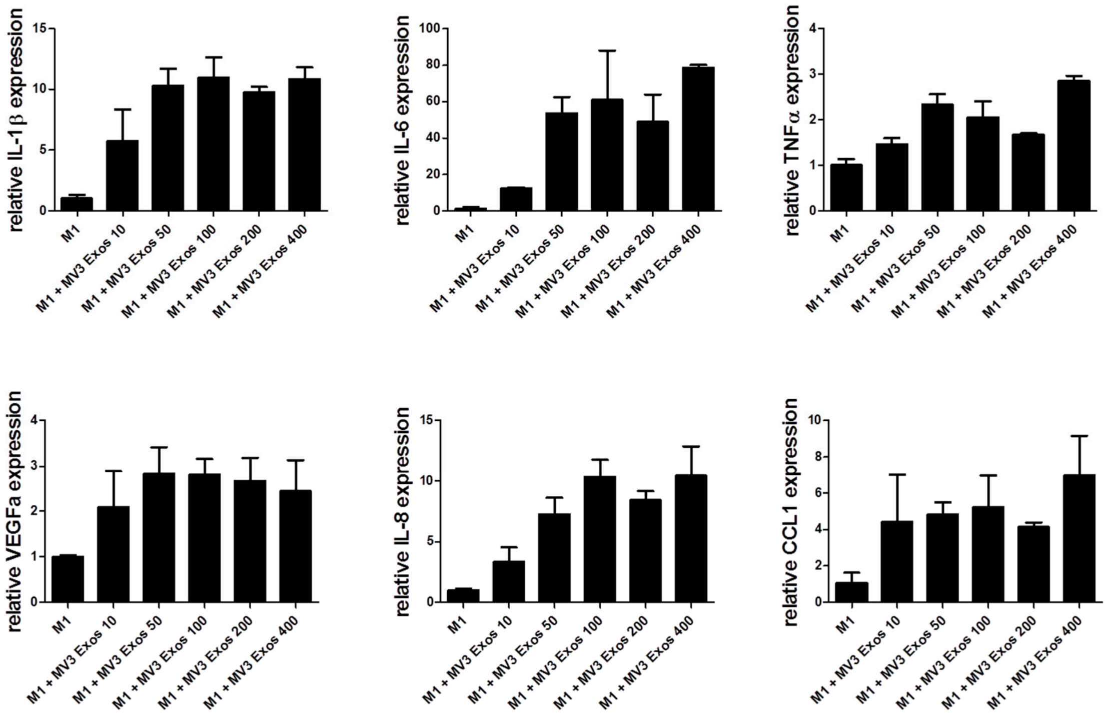

Supplementary Figure 1. Induction of macrophages gene expression by melanoma derived exosomes. (A) Heatmap shows gene expression profile of M1 and M2 polarized THP-1 cell derived macrophages in comparison to M1 macrophages treated with exosomes of 3 different melanoma cell lines (MV3, WM35, Wm902B). Heatmap represents the log2 fold change of qRT-PCR analyses. qRT-PCR analyses for IL-1β, IL-6, IL-8, VEGFa, TNFα and CCL1in M1 macrophages. (B) M1 macrophages were treated with 25 µg/ml MV3 derived exosomes for the indicated time. (C) M1 macrophages were treated with indicated concentrations of MV3 derived exosomes (µg/ml) for 12h. Bars represent the mean ± standard deviation of at least three independent experiments.

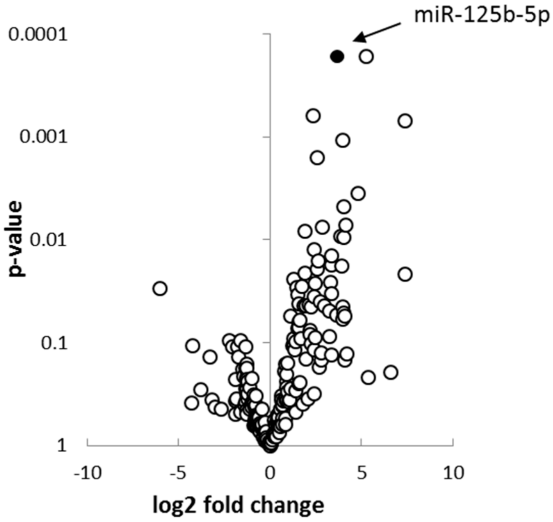

Supplementary Figure 2. miR-125b-5p expression is increased in melanoma cells. Volcano plot shows miRNA enrichment of miR-125b-5p in melanoma cell lines compared (WM9, WM35, WM902B) to NHEMs.

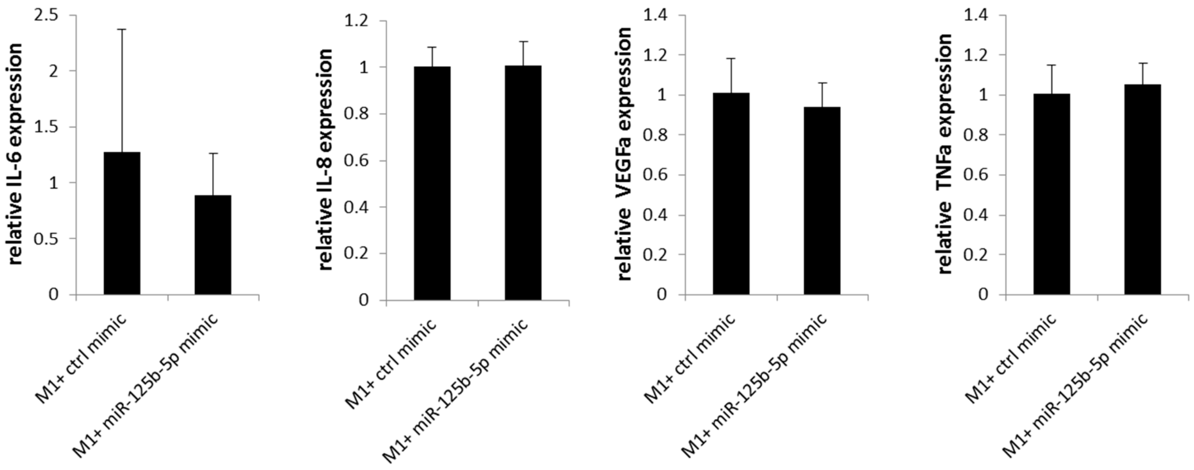

Supplementary Figure 3. IL-6, IL-8, VEGFa and TNFα expression is not induced by miR-125b-5p overexpression. qRT-PCR analysis for the expression of IL-6, IL-8, VEGFa and TNFα in M1 macrophages transfected with control mimics or miR-125b-5p mimics (48h). Bars represent the mean ± standard deviation of at least three independent experiments.
